# Oxidative Stress Linked Organ Lipid Hydroperoxidation and Dysregulation in Mouse Model of Nonalcoholic Steatohepatitis: Revealed by Lipidomic Profiling of Liver and Kidney

**DOI:** 10.3390/antiox10101602

**Published:** 2021-10-12

**Authors:** Yue Wu, Zhen Chen, Hirotoshi Fuda, Takayuki Tsukui, Xunzhi Wu, Nianqiu Shen, Natsuki Saito, Hitoshi Chiba, Shu-Ping Hui

**Affiliations:** 1 Faculty of Health Sciences, Hokkaido University, Kita-12, Nishi-5, Kita-Ku, Sapporo 060-0812, Japan; wuyue123@hs.hokudai.ac.jp (Y.W.); chenzhen@hs.hokudai.ac.jp (Z.C.); hfuda@hs.hokudai.ac.jp (H.F.); xunzhi.wu.m6@elms.hokudai.ac.jp (X.W.); nianqiu.shen.z0@elms.hokudai.ac.jp (N.S.); saito.natsuki.e9@elms.hokudai.ac.jp (N.S.); 2 Department of Nutrition, Sapporo University of Health Sciences, Nakanuma Nishi-4-2-1-15, Higashi-Ku, Sapporo 007-0894, Japan; tsukui@sapporo-hokeniryou-u.ac.jp (T.T.); chiba-h@sapporo-hokeniryou-u.ac.jp (H.C.)

**Keywords:** nonalcoholic steatohepatitis, lipidomics, oxidative stress, lipid hydroperoxides, triglycerides, ethanolamine phospholipids, plasmalogens, cardiolipins, fatty acyls, molecular species

## Abstract

Nonalcoholic steatohepatitis (NASH) is a prevalent disease related to lipid metabolism disorder and oxidative stress. Lipid hydroperoxidation is known to be a critical driving force of various disorders and diseases. However, the combination of both intact and hydroperoxidized lipids in NASH has not yet been studied. In this work, the liver and kidney samples from NASH-model mice were comprehensively investigated by using the LC/MS-based lipidomic analysis. As a result, triglycerides showed the amount accumulation and the profile alteration for the intact lipids in the NASH group, while phosphatidylethanolamines, lysophosphatidylethanolamines, plasmalogens, and cardiolipins largely depleted, suggesting biomembrane damage and mitochondria dysfunction. Notably, the lipid hydroperoxide species of triglyceride and phosphatidylcholine exhibited a significant elevation in both the liver and the kidney of the NASH group and showed considerable diagnostic ability. Furthermore, the relationship was revealed between the lipid metabolism disturbance and the lipid hydroperoxide accumulation, which played a key role in the vicious circle of NASH. The present study suggested that the omics approach to the lipid hydroperoxide profile might be the potential diagnostic marker of NASH and other oxidative stress-related diseases, as well as the evaluative treatment index of antioxidants.

## 1. Introduction

Nonalcoholic fatty liver disease (NAFLD) is a prevalent noninfectious chronic liver disease with an estimated global occurrence of about 20–30%, becoming one of the most important causes of liver disease worldwide [1,2,3]. This global burden, which is intimately due to an unhealthy lifestyle, represents simple steatosis to steatohepatitis, cirrhosis, and even hepatocellular carcinoma [1,4]. Nonalcoholic steatohepatitis (NASH), as the histological phenotype of NAFLD, is associated with structural and functional liver modifications, including steatosis, lobular inflammation, and hepatocyte ballooning of the liver. Moreover, acting as a multifactorial and progressive disease, NASH is often accompanied by comorbidities such as obesity, insulin resistance, dyslipidemia, and chronic kidney disease (CKD) [5,6]. The underlying mechanism for the development of NASH is complex with a variety of factors, which has been described as “multiple-hit” (as the replacement of the previous “two-hit”) in recent years, such as hepatic steatosis, oxidative stress, apoptosis, inflammation, and gut-derived endotoxin [7,8]. Nevertheless, until the present, the elucidation of the entire mechanism of NASH, especially at the molecular level, remains uncovered.

Oxidative stress has been recognized as one of the key factors in NASH development, which originates from hyperleptinemia-induced oxidation of hepatic lipids and leads to the lipotoxicity of organs and release of the reactive oxygen species (ROS) [8]. At the same time, oxidative stress, considered a major factor in chronic liver diseases, triggers hepatocytes’ stress pathways and induces inflammation and fibrogenesis, contributing to the progression of NASH [9]. Moreover, the oxidative damage to the mitochondria results in mitochondrial dysfunction, weakening the *β*-oxidation of lipids, causing abnormal lipid accumulation, and eventually worsening the oxidative stress-antioxidation balance [10]. Therefore, a variety of antioxidants have been applied to treat NASH/NAFLD, relieving symptoms and improving metabolism [7]. On the other hand, the changes of lipidome in NASH have been gradually revealed, which has been claimed to associate with genes, proteins, and other metabolites [11,12]. The key findings mainly included fatty acids, especially polyunsaturated fatty acids (PUFA), glycerolipids and glycerophospholipids with molecular diversity, and fatty acid oxidation products [11], though there might be more characteristic alterations. As indirect evidence, the level of malondialdehyde (MDA), which serves as the end product of lipid hydroperoxidation and can be measured by thiobarbituric acid-reactive substances (TBARS) assay, has also been applied on NASH or NASH-model studies [13,14]. Importantly, the lipid hydroperoxides are known as the crucial intermediates of oxidative reactions [15], and the ongoing exploration can strengthen our understanding of oxidative stress-associated disease states, for example, NASH. However, studies which investigate NASH-induced lipidomic changes that include both intact and hydroperoxidized lipids by direct measurement are quite rare.

Therefore, in the present work, the NASH model mice were used to conduct a comprehensive lipidomic analysis covering both intact lipids and lipid hydroperoxides in the liver and kidney tissues using liquid chromatography-mass spectrometry (LC/MS). The diversity and featured differences were revealed between the normal and the NASH model groups with organ specificity. Furthermore, the usefulness of lipid hydroperoxides as a feasible biomarker for NASH diagnosis was evaluated. The correlation between lipid hydroperoxide accumulation and intact lipid dysregulation was also discussed.

## 2. Materials and Methods

### 2.1. Chemicals

For the authentic internal standards (IS), triacylglycerol (TG) 11:0/11:0/11:0 and free fatty acid (FFA) 17:0 were obtained from Sigma-Aldrich (St. Louis, MO, USA), and phosphatidylcholine (PC) 13:0/13:0, phosphatidylethanolamine (PE) 15:0/15:0, phosphatidylinositol (PI) 8:0/8:0, lysophosphatidylcholine (LPC) 15:0, lysophosphatidylethanolamine (LPE) 13:0, and lysophosphatidylinositol (LPI) 13:0 were purchased from Avanti Polar Lipids (Alabaster, AL, USA). Cardiolipin (CL) 15:0/15:0/15:0/15:0 was synthesized in our laboratory previously [16]. Other chemicals and reagents, which were of the highest grade available, were purchased from Sigma-Aldrich unless specified. The mixed solution of all the IS was prepared with methanol (containing 0.006% butylated hydroxytoluene, *w/v*) and stored at −80 °C for no more than two weeks before use.

### 2.2. Animals and NASH Modeling

All animal protocols conformed to the Public Health Service Guide for Care and Use of Laboratory Animals, which were approved by (1) Institutional Animal Care and Use Committee at Hokkaido University (Approval No. 10-0028) and (2) the ethical committee at the Faculty of Health Sciences, Hokkaido University (Approval No.14-76). The study design complied with the associated guidelines and 3R (Replacement, Reduction, and Refinement) principle. In detail, the NASH model in C57BL/6J mice was induced with a high-fat diet and oxidized low-density lipoprotein injection as previously described [17,18]. The detailed information on animal feeding and modeling is described in Supplementary file 1. The mice of both normal and NASH-modeling groups (*n* = 8 for each) were fasting for 12 h before being anesthetized and sacrificed. The model was considered established successfully based on the physiological, histological, and blood data, which were reported in our previous work [18]. The collected liver and kidney samples were snap-frozen immediately in liquid nitrogen and stored at −80 °C until analysis.

### 2.3. Tissue Sample Preparation and Lipid Extraction

Total lipids from tissue samples were extracted according to Folch’s method [19] as previously described [16]. In brief, the homogenized tissue sample was extracted with ice-cold chloroform/methanol 2:1 (*v/v*, with IS). The extraction was performed twice, and the combined extract was dried under vacuum. The obtained total lipids were dissolved in methanol and centrifuged to remove any insoluble material prior to analysis. The sample pretreatment was completed within 1 h to avoid lipid auto-oxidation or degradation.

### 2.4. LC/MS Parameters and Data Processing

The lipidomic analysis and semi-quantitation were carried out on a Shimadzu Prominence HPLC (Shimadzu Corp., Kyoto, Japan) and an LTQ Orbitrap mass spectrometer (Thermo-Fisher Scientific Inc., San Jose, CA, USA) operated in both electrospray ionization (ESI) positive and negative ionization modes, which was accorded to our previous studies [16,20]. In brief, the chromatographic conditions were as follows: Column, Atlantic T3 C18 (2.1 mm × 150 mm, 3 mm, Waters, Milford, MA, USA); mobile phase, water with 10 mM ammonium acetate (A), isopropanol (B), and methanol (C); column oven temperature, 40 °C; and flow rate, 200 mL/min. The MS scan parameters were as follows: HR-MS^1^ scan mode, Fourier transform (FT); resolution power, 60,000; scan range, *m/z* 150–1100 under positive mode and *m/z* 220–1650 for negative mode; and MS^2^ scan mode: Ion-trap (IT), using collision-induced dissociation (CID) by data-dependent acquisition, with the normalized collision energy set at 35% and the isolation width set at 2 Da. The sample injection sequence was randomized to minimize the running time-induced variation.

The raw data were processed by using the workstation Xcalibur 2.3 (Thermo Fisher Scientific, Inc.). The mass tolerance was 5.0 ppm for HR-MS^1^ and 0.5 Da for MS^2^. The identification of intact and hydroperoxidized lipids was accorded to the retention behavior in reversed-phase LC, the HR-MS^1^, and the MS^2^ fragments, with the help of comparison with LIPIDMAPS (www.lipidmaps.org, accessed on 12 December 2020) as well as our in-house library [16,20]. The annotated lipid species were indicated as follows: “lipid class + the number of acyl carbon atoms + the number of acyl double bonds”. For semi-quantitation, the peak of the extracted ion chromatogram for each lipid species was integrated, and the amount of each lipid species was calculated as the equation below:

Amount _analyte_ = Amount _IS_ × Peak area _Analyte_ / Peak area _IS_

### 2.5. Statistics

All the data were expressed as the mean and standard deviation (SD). Two-tailed Student’s *t*-test were calculated using SPSS 24 (IBM, Chicago, IL, USA), of which the differences were considered significant at *p* < 0.05. Principal component analysis (PCA) was performed by JMP pro 16 (SAS Institute, Cary, NC, USA), and orthogonal partial least squares discriminant analysis (OPLS-DA) was processed using SIMCA-P 14.1 (Umetrics, Umeå, Sweden). The diagnostic performance was evaluated based on the receiver operating characteristic (ROC) using Prism 8.0 (GraphPad Software, La Jolla, CA, USA). The correlations between variables were assessed by using R 4.0 (www.r-project.org, accessed on 31 March 2021), and the lipidomic network was visualized using Cytoscape 3.8 (cytoscape.org, accessed on 8 May 2021).

## 3. Results and Discussion

### 3.1. Diversity of Intact Lipids between Tissues of Normal and NASH Model

The present LC/MS and MS/MS analysis resulted in the annotation of 270 intact lipid molecular species based on their retention behavior under the current chromatography condition, along with their protonated ([M + H]^+^, for PC and LPC), deprotonated ([M − H]^−^, for FFA, PE, PlsEtn, LPE, PI, and LPI), ammoniated ([M + NH_4_]^+^, for TG), or acetate ([M + Ac]^–^, for PlsCho) ion peaks on HR-MS, which were further identified according to the fragmentation of MS/MS (shown in Table S1 of Supplementary file 2). At the same time, 18 hydroperoxidized lipid species were detected, including 9 hydroperoxides of TG (TGOOH) and 9 hydroperoxides of PC (PCOOH) (shown in Table S2 of Supplementary file 2).

**Figure 1:**
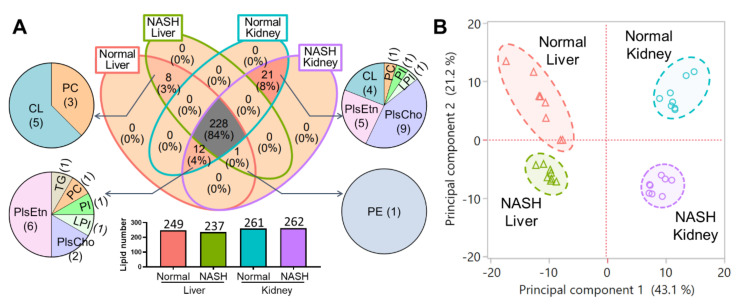
(**A**) Comparison for the diversity of the intact lipids among four groups: Normal liver (249), NASH liver (237), Normal kidney (261), and NASH kidney (262). Numbers represent the detected lipids in the sample. (**B**) Score plot of PCA revealed the distinguished profile of the intact lipids in liver/kidney tissues and normal/NASH groups.

Among the annotated intact lipids, there were 228 species detected from both the liver and the kidney for normal or NASH groups, which accounted for 84% of the whole detected intact lipids. Additionally, eight species existed only in the liver, which comprised PC and CL, while 21 species were uniquely in the kidney, which mainly consisted of plasmalogens for both choline (PlsCho) and ethanolamine (PlsEtn) types, as well as CL (Figure 1A). Specifically, the kidney (of both normal and NASH) showed more complex plasmalogen varieties (15 PlsCho and 24 PlsEtn), higher than the normal liver (6 PlsCho and 19 PlsEtn) and the NASH liver (4 PlsCho and 13 PlsEtn). These results indicate that the kidney exhibited a higher lipid diversity than the liver. Interestingly, there were 12 lipids detected in the kidney and the normal liver samples, but not the NASH liver, which suggested that the lipid diversity varied along with both tissue specificity and normal/NASH distinction.

Moreover, PCA was performed using all the intact lipid species as variables (Figure 1B; eigenvalues and loading plot are shown in Figure S1 of Supplementary file 2), of which the first two principal components accounted for 43.1% and 21.2% of the total variation, indicating the complexity of the whole variance in these samples. The distinctive clustering of the four groups was revealed clearly, suggesting the characterized distinctions of the whole intact lipid profile between liver and kidney and between normal and NASH. Interestingly, the first principal component represented the organs, while the second principal component indicated the model states. Although the pathogeneses of NASH are known to be connected with liver inflammation and hepatocyte damage [21], in this study, the corresponded changes were also reflected in the kidney. Therefore, the perturbations of the lipidomic profiles, and the dissimilarities of the changes between liver and kidney, were worthy of further comparison.

### 3.2. Tissue-Specific Changes of Intact Lipid Species in NASH Mouse Model

**Figure 2:**
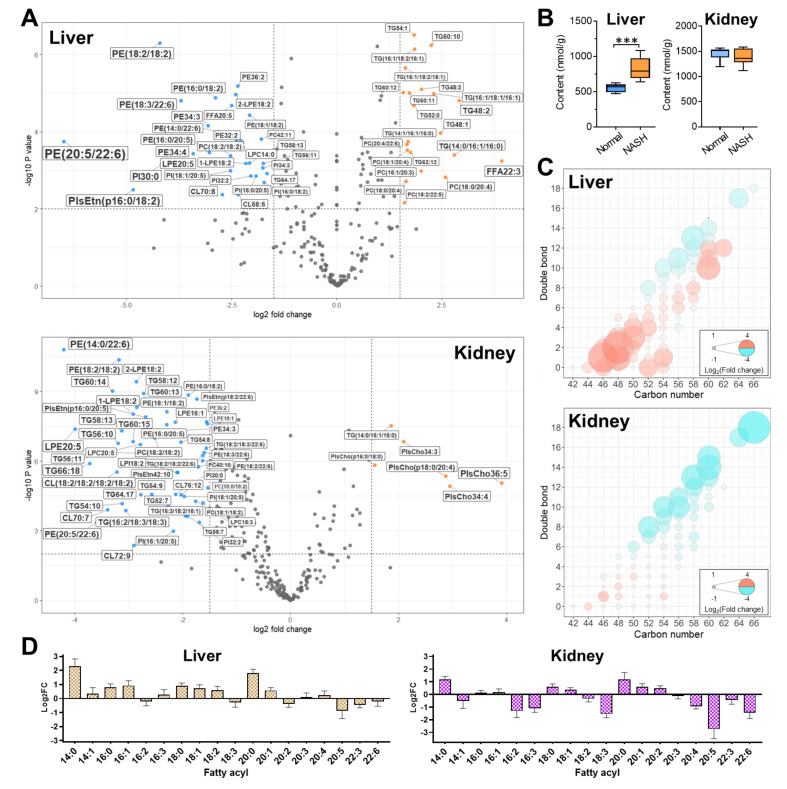
(**A**) Volcano plots for the changes of the lipids in liver and kidney between normal and NASH groups. Significance was defined with both *p* < 0.01 and |log_2_(fold change)| > 1.5, and the significantly changed lipids are indicated in orange (increased in NASH group) or blue (decreased in NASH group). (**B**) Comparison of total TG between the normal and the NASH groups in liver and kidney. *** *p* < 0.001, calculated by two-tailed *t*-test. (**C**) Bubble plots of TG profile changes in the liver and the kidney in the NASH group. The color (ranging from red to cyan) and the bubble scales represent the degree of the difference, calculated as log_2_(fold change of NASH to normal). (**D**) The changes for the fatty acyl composition of TG in NASH liver and kidney.

The volcano plots have been performed to explore the most significant changes of all the intact lipid species in the liver and the kidney samples between normal and NASH groups (Figure 2A), in which 51 differentially lipids (both *p*-value < 0.01 and |log_2_(fold change)| > 1.5) were found in the NASH liver, and 57 species were found in the NASH kidney, respectively. Notably, the alteration for the NASH model of intact lipids differentiated between liver and kidney: in the liver, 21 species increased, and 30 species decreased; on the other hand, in the kidney, only 6 lipids increased, while the other 51 species, as most of the differences, showed significant decreasing.

#### 3.2.1. TG Accumulation in NASH Liver and Profile Alteration in NASH Kidney

About one-third of the significantly altered lipid species in both liver and kidney belonged to TG; however, the detailed alterations differed between the two organs. In the NASH model mice, TG was found significantly accumulated in the liver samples (823.8 ± 155.9 nmol/g), accounting for 1.5-fold of the normal (554.5 ± 58.4 nmol/g) (Figure 2B), which was in agreement with previous studies [22,23]. The increased TG was considered to store in lipid droplets of hepatocytes, which was reported in NASH model mice by Amrutkar et al. [24]. In contrast, there was no such difference for the kidney (Normal, 1462.5 nmol/g; NASH, 1382.2 nmol/g). Taking consideration of the molecular composition, as Figure 2C shows, the total TG accumulation in the NASH liver was predominantly due to the accretion of the more saturated species, especially those with shorter carbon chain length (i.e., 44–54) and fewer double bonds (i.e., 0–4). Even for the longer chain (e.g., C60) TG species, the changing trends depended on double bonds: those with fewer double bonds increased, and those with more double bonds decreased. In the NASH kidney, the most outstanding alterations were the reduction in the longer chain highly unsaturated species (i.e., 58–66 carbons with 12–18 double bonds). Furthermore, with regard to the fatty acyl composition in TG, the consistent results of the alterations were obtained, in which the most apparent alterations included the increase in C14:0, C16:0, C18:0, and C20:0 in the NASH liver, and the decrease in C18:3, C20:5, and C22:6 in the NASH kidney (Figure 2D). To the best of our knowledge, this was the first report on the distinguishment of TG profile among NASH organs. The long-chain polyunsaturated fatty acids (PUFA) are known to prevent steatosis, inflammation, and dyslipidemia, and reduce the risk of carcinogenesis in NASH [23]. There have been clinical reports on the increasing TG (especially for the shorter and saturated species) in the liver of NASH patients [25,26,27], which supported the present results. Studies on the other severe alteration, i.e., the diminishing of long-chain PUFA-enriched TG in the NASH kidney, are still rare.

#### 3.2.2. NASH Model Induced the Depletion of Functional Ethanolamine Phospholipids

**Figure 3:**
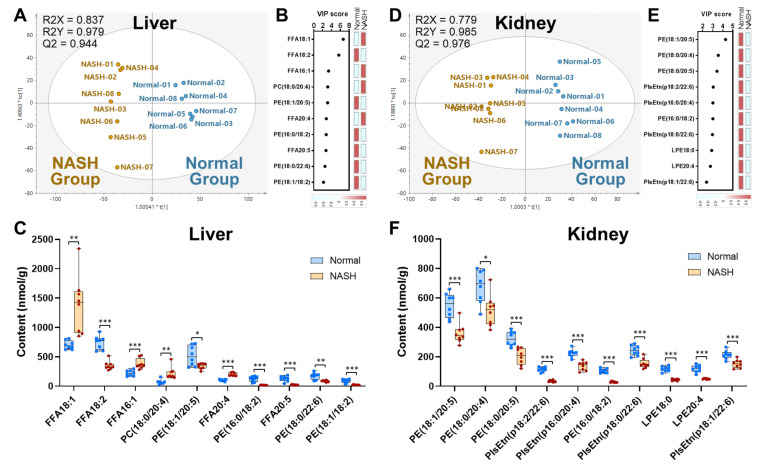
OPLS-DA of intact lipidomic characteristics in liver and kidney, including scatter plots (**A**,**D**), VIP ranking (**B**,**E**), and the amount comparison of the selected lipids (**C**,**F**) together with the VIP scores of the most significant variables. * *p* < 0.05, ** *p* < 0.01, *** *p* < 0.001, calculated by two-tailed *t*-test.

To achieve more insightful discrimination of lipid metabolic impairments in the NASH mouse model, the OPLS-DA was accomplished for liver and kidney samples. Regardless of liver or kidney, the normal and the NASH samples were clearly separated into two clusters in the scatter plots (Figure 3A,D), suggesting their distinct lipid profiles. Then, the top 10 significant variables as lipid species driving separation of the clusters were attained for liver and kidney, respectively, based on their variable of importance (VIP) scores (Figure 3B,E). Subsequently, the content of these potential markers was compared between the normal and the NASH groups (Figure 3C,F). In detail, the potential lipid markers included 5 FFA, 4 PE, and 1 PC for the liver, and 4 PE, 4 PlsEtn, and 2 LPE for the kidney. It is notable that all these lipids in the kidney were decreased, which drew our attention.

Among the top varied lipids in the NASH kidney samples, all the PE species were found to contain PUFA (e.g., C20:4 and C20:5), which was in agreement with the renal TG profile alteration, as mentioned above. These renal PE were decreased by 24.1–73.1% in the NASH group (*p* < 0.05 for all), which was also similar in the liver. As the main biomembrane constituent, PE is functionally associated with various cellular processes, such as oxidative phosphorylation, membrane fusion [28], and even the maintenance of mitochondrial morphology and function [29]. The reduction in PE has been proven to introduce biomembrane damage and cellular apoptosis [30]; the resulted disorder of the PC/PE ratio even causes severe organ lipid metabolism disorders [31]. Therefore, our results of the PE depletion might suggest the occurrence of kidney dysfunction in the NASH group from the view of lipidome. In addition, the PUFA-enriched PlsEtn species was significantly reduced by 28.8–70.0%. These unique ethanolamine phospholipids with vinyl-ether linkage, also known as plasmalogen, are reported to participate in multiple physiological activities, including cell membrane maintenance and signal transduction; moreover, they protect against oxidative stress and inflammation [32]. The depletion of PlsEtn could thus indicate the oxidation-induced damage of functional phospholipids in the kidney of the current NASH model mice. Interestingly, these depletions were found only for the ethanolamine plasmalogen but not for the choline plasmalogen. We also noticed that the deacylated products of ethanolamine phospholipids (including both PE and PlsEtn), i.e., LPE species, showed large depletion in NASH kidney (*p* < 0.001 for both LPE18:0 and LPE20:4). Similar findings of the decreased LPE were reported previously in serum and liver from NASH and NAFLD patients [33,34], for which one of the reasons was the increase in lysophosphatidylethanolamine acyltransferase 1 (LPEAT 1) [34]. According to our data, not only LPE but also PE and PlsEtn decreased in the NASH samples, indicating the deficiency of the de novo synthesis from ethanolamine to phospholipids. It has been demonstrated that the CDP-ethanolamine pathway for utilizing ethanolamine and biosynthesizing PE occurs in the endoplasmic reticulum (ER) [35], while the ER stress, triggered by cellular lipid accumulation, has been found in NAFLD, followed by the depletion of PE [36]. Another important pathway for PE biosynthesis is the phosphatidylserine decarboxylase (PSD) pathway, which exists exclusively on the outer aspect of mitochondrial inner membranes [31]. Therefore, the depletion of ethanolamine phospholipids found in the current study supported the viewpoint of abnormalities of ER and mitochondria in NASH [37,38].

#### 3.2.3. Reduction in CL Associated with Mitochondria Dysfunction in NASH Model Mice

**Figure 4:**
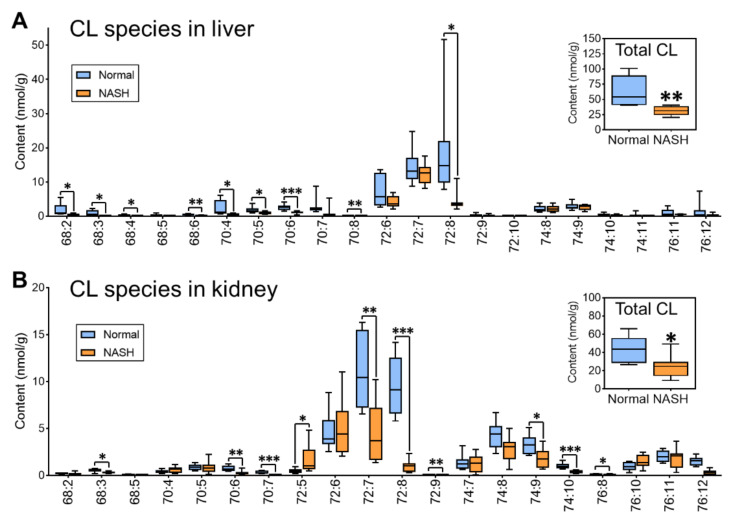
Comparison of the CL species and the total CL between normal and NASH groups in the liver (**A**) and the kidney (**B**). * *p* < 0.05, ** *p* < 0.01, *** *p* < 0.001, calculated by two-tailed *t*-test.

Apart from the depletion of functional ethanolamine phospholipids, we also noticed that another important class of phospholipids, CL, exhibited a large reduction in both the liver and the kidney of the NASH group. For the total CL, the liver and the kidney of the NASH group showed 49.9% (63.0 nmol/g vs. 31.6 nmol/g) and 43.0% (43.5 nmol/g vs. 24.8 nmol/g) reduction, respectively, compared with the normal groups (Figure 4). These mitochondria-specific lipids are known to maintain the mitochondrial function and dynamics and thus associate with various pathophysiological situations and diseases [39,40]. Thus, our results of total CL content suggested a severe loss of mitochondrial function for the NASH group in both liver and kidney, which were often considered as the mitochondrial injury relative to the oxidative damage. Taking a closer look at individual CL species in both the liver and kidney, we noticed that the predominant species were CL72:6 (18:1/18:2/18:2/18:1), CL72:7 (18:1/18:2/18:2/18:2), and CL72:8 (18:2/18:2/18:2/18:2). These C18:2-enriched CL species, serving as the mature CL, play an essential role in mitochondrial function [41]. In the current study, these CL species exhibited an even more serious depletion, especially CL72:8, which lost 77.4% (18.8 nmol/g vs. 4.3 nmol/g) in the liver and 89.3% (9.4 nmol/g vs. 1.0 nmol/g) in the kidney. It has been summarized that mitochondria play an essential role in the pathogenesis of NAFLD [42], and mitochondrial dysfunction is associated with the loss of CL content and the alterations in the fatty acyl composition [41]. Thus, our results revealed that the deterioration of both CL quantity (as the total CL content) and CL quality (as the mature CL content) occurred in both liver and kidney of NASH, which was closely related to damage of mitochondria. This not only impaired the fat homeostasis but also the mitochondrial dysfunction, resulting in the overproduction of ROS that prompts lethal hepatocyte injury associated with NAFLD [43].

### 3.3. Accumulation of Oxidized Lipid Species in Liver and Kidney of NASH Model Mice

**Figure 5:**
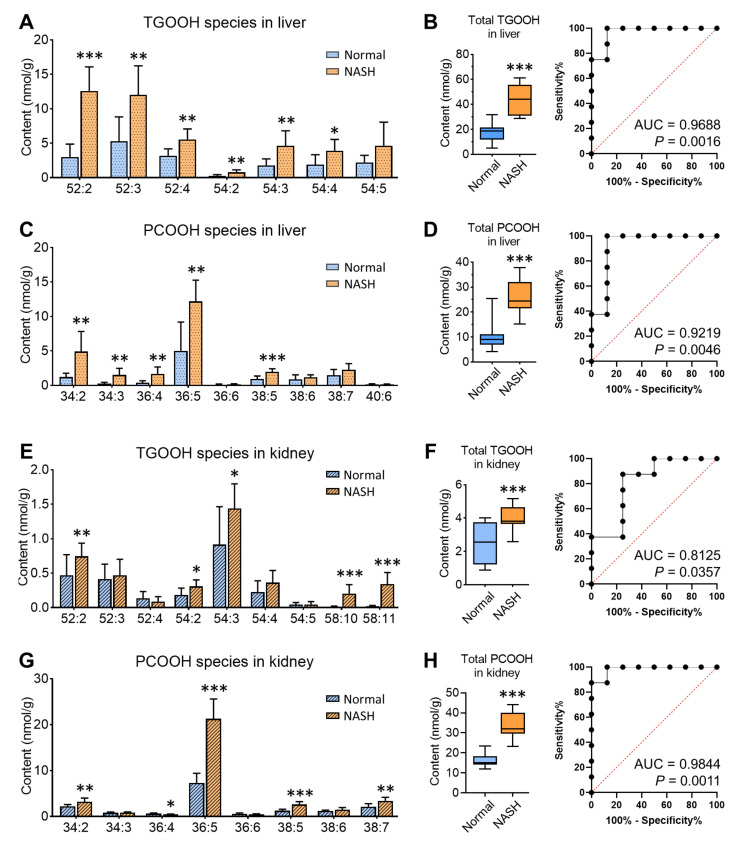
Accumulation of lipid hydroperoxides in NASH model mice. (**A**) TGOOH species in the liver. (**B**) Comparison and the ROC curve of the total TGOOH in the liver. (**C**) PCOOH species in the liver. (**D**) Comparison and the ROC curve of the total PCOOH in the liver. (**E**) TGOOH species in the kidney. (**F**) Comparison and the ROC curve of the total TGOOH in the kidney. (**G**) PCOOH species in the kidney. (**H**) Comparison and the ROC curve of the total PCOOH in the kidney. * *p* < 0.05, ** *p* < 0.01, *** *p* < 0.001 vs. normal, calculated by two-tailed *t*-test.

Besides the intact lipid species, the hydroperoxidized lipids were also measured, including the hydroperoxides of TG (TGOOH) and PC (PCOOH) in our study; 7 TGOOH and 9 PCOOH species were detected in the liver, and 9 TGOOH and 8 PCOOH species were detected in the kidney. The comparison of their content between normal and NASH is shown in Figure 5A,C,E,G, respectively, in which most of the species exhibited significantly elevated levels. For TGOOH, the predominant species in the NASH liver were 52:2 (28.7% ± 2.2%) and 52:3 (27.1% ± 4.1%), and in the NASH kidney was 54:3 (35.8% ± 5.2%), suggesting that the TGOOH profile might be related to the intact TG profile. Moreover, TGOOH content in the liver (the average: 44.2 ± 12.6 nmol/g) was much higher than in the kidney (the average: 4.0 ± 0.8 nmol/g) of the NASH group, indicating the accordance between the intact TG accumulation and the TGOOH accumulation. While for PCOOH, the dominant species in both liver and kidney of NASH was 36:5 (48.2% ± 9.0% for the liver; 63.1% ± 1.8% for the kidney). Notably, we found that there seemed no specific accumulation pattern among different species. Therefore, we calculated the total TGOOH and PCOOH as the sum of every species. In the NASH group, TGOOH elevated 2.5-fold in the liver and 1.7-fold in the kidney, while PCOOH elevated 2.5-fold and 2.1-fold, respectively (*p* < 0.001 for all of them).

Considering that these lipid hydroperoxides are supposed to be the key intermediates playing a role in the pathogenesis of NASH and its relevant complications, we subsequently evaluated the diagnostic powers of total TGOOH and total PCOOH as potential NASH markers (Figure 5B,D,F,H). All the ROC curves showed that lipid hydroperoxides expressed promising capabilities, with the area under curve (AUC) more than 0.80 and *p* values less than 0.05. Interestingly, for the liver, total TGOOH (AUC: 0.9688, *p* = 0.0016) showed stronger predictive ability than PCOOH (AUC: 0.9219, *p* = 0.0046), while for the kidney, PCOOH (AUC: 0.9844, *p* = 0.0011) was more reliable than TGOOH (AUC: 0.8125, *p* = 0.0357). The possible explanation might be due to the characteristic changes of specific intact lipid classes: TGOOH originates from the excessive intact TG stored in lipid droplets, which was attacked by the produced ROS under oxidative stress; while PCOOH as the hydroperoxidation products of the major constitutes of biomembrane, PC, are more closely involved in the damage and loss of the phospholipids. Nevertheless, the detailed differences of their production mechanisms and their interactions remain to be explored.

**Figure 6:**
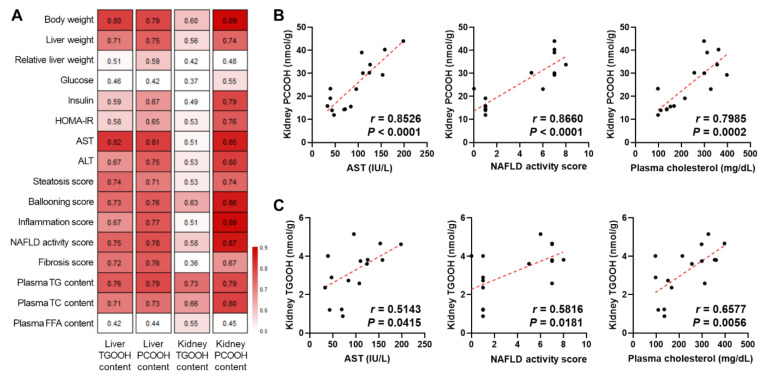
(**A**) Heatmap of the Pearson correlation coefficients between the organ lipid hydroperoxides and the physiological, histological, and blood data. (**B**) Scatter plots of kidney total PCOOH content and representative NASH-related biological indexes. (**C**) Scatter plots of kidney total TGOOH content and representative NASH-related biological indexes.

For clinical descriptions and evaluations of NASH, the commonly used approaches include body examinations, blood tests, liver biopsy, and others. Therefore, we investigated the possible usefulness of the hepatic and renal lipid hydroperoxides as a diagnostic standard of NASH. The correlations between the organ lipid hydroperoxides obtained in this study, and the physiological, histological, and blood data obtained in our previous study [18], were calculated as Pearson’s correlation coefficient (Figures S2–S5 of Supplementary file 2). As summarized in the heatmap of Figure 6A, clear positive correlations were observed, suggesting the consistency of lipid peroxidation in the organs and a series of pathological alterations in the liver and blood. Notably, the kidney PCOOH showed the strongest correlation with the biological indexes over other lipid hydroperoxides (Figure 6B), followed by the liver PCOOH and the liver TGOOH, which behaved with a similar relevancy. In contrast, the kidney TGOOH showed the weakest correlation (Figure 6C). These results also suggested it might be possible to estimate the lipid peroxidation degree using certain biological data; the lipid hydroperoxides were indeed intimately linked to NASH.

**Figure 7:**
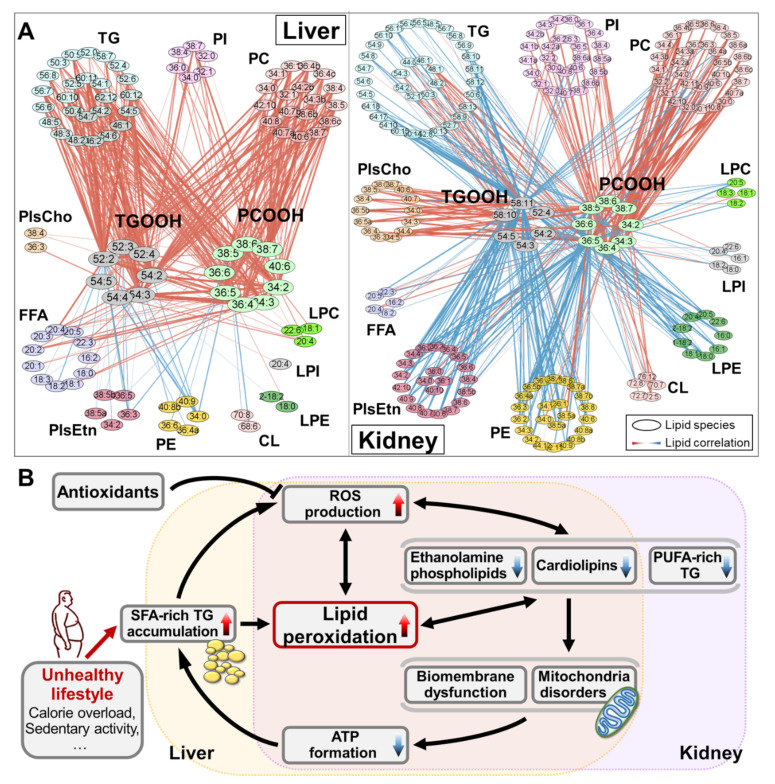
(**A**) Visualized connective network of PCOOH, TGOOH, and intact lipid species in the liver (left) and the kidney (right) of the NASH model mice. The colors of the nodes represent different lipid classes, and the color and width of the lines connecting two lipid species correspond to their dependency as Pearson’s coefficient of correlation. (**B**) Putative process of the “vicious circle” for oxidative stress associated with lipid dysregulation in NASH.

In order to obtain a global view on the relationships between all these intact and hydroperoxidized lipid species in the NASH model mice, we constructed the correlation-based dependency network for the liver and the kidney (Figure 7A). In both organs, the dominant connections were revealed as TGOOH and PCOOH positively associated with PC. For the liver, the intact TG and PC species were clearly connected to these lipid hydroperoxides, especially for the connection of TG-TGOOH, which suggested that the accumulation and the subsequent oxidation of TG in NASH, as aforementioned. While in the kidney, the most distinguished connections were the negative correlation appeared in ethanolamine phospholipids (PE, PlsEtn, and LPE), indicating the relationship between the depletion of membrane-functioned lipids, which lead to mitochondrial dysfunction and membrane damage, with the production of lipids hydroperoxides. Therefore, the accumulation of lipid hydroperoxides might help to reveal the NAFLD-induced hepatic steatosis and hepatitis as the syndrome of NASH, and the CKD or even renal insufficiency. In short, the comprehensive omics data could help us better understand the molecular mechanisms underlying NASH and lipid oxidation at the molecular level.

Therefore, the putative processes of oxidative stress and lipid dysregulation in NASH were proposed as the “vicious circle” shown in Figure 7B. Our results suggested that the accumulation of TG (mainly those enriched with shorter and more saturated fatty acyls) became a heavy burden to the organs (especially for the liver) and aggravated the imbalance between pro-oxidant and antioxidant species. As a result, the lipid hydroperoxides (e.g., TGOOH and PCOOH revealed in this study) accumulated, together with the hydroperoxidation products (e.g., MDA [44] and 4-HNE [45]), and the ROS excessively produced. Such oxidation products seriously influenced the structural and functional normality of lipid-consisted biomembrane and contributed to the pathological processes of NASH [7]. Moreover, in return, the accelerated generation of ROS worsened the oxidative degree to the body. Similar trends of the elevated ROS levels, along with the lipid oxidation, were demonstrated not only in the organs of NAFLD/NASH [46,47], but also of other metabolic syndromes, such as diabetes mellitus [16,48], hypertension [49,50], CKD [51,52], and atherosclerosis [53]. Furthermore, the deficiency of the physiological lipids, particularly PUFA, ethanolamine phospholipids, and CL, leads to biomembrane dysfunction-related disturbances, especially mitochondrial dysfunction, which could oppositely cause free radical production and lipid peroxidation [29,54]. As the result of mitochondrial imperfection, the utility of substance (lipids) and the production of energy (ATP) was impeded, eventually making the problem of lipid overloading even more severe, closing the vicious circle. Furthermore, despite the hepatic lipid metabolism disturbance, the kidney also suffered similar problems from the view of lipidomic changes; additionally, PUFAs were deficient, being the kidney-characterized feature. All these renal metabolic pathologies serve as CKD development factors; however, the organ/tissue-specific assays combining genome, transcriptome, and proteome are needed to study in the future. In addition, the supplementation of antioxidants, whether natural or artificial, are considered to intervene in this progression, improving the hepatic and renal lipidome, especially suppressing the accumulation of lipid hydroperoxides.

Considering the central role of oxidative stress and peroxidation of the lipids, we proposed that the lipid hydroperoxide levels might be potentially designated as the diagnostic standards of NAFLD/NASH and other oxidative stress-related chronic metabolic diseases. The quantitative monitoring of the lipid hydroperoxides was also hopeful to be the evaluation index for the effects of antioxidants as a therapeutic strategy. Thus, the validation of the lipid hydroperoxide profile analysis by performing the antioxidant treatment trials (by using naturally produced or artificially designed antioxidative agents) required further investigation. Another limitation of this work was that the sample size was relatively small, which limited the generalizability of these findings. Future studies need to develop the quantitative determination of these lipid hydroperoxides and combine their data with multidimensional biological, clinical, and pathological indexes in the larger cohorts. Nevertheless, the comprehensive assessment of both the intact and the hydroperoxidized lipid profiles took an exploratory step for understanding the key roles of oxidative stress-involved lipid species, particularly lipid hydroperoxides, in the mechanisms of NAFLD/NASH.

## 4. Conclusions

In conclusion, the current LC/MS-based lipidomic study on NASH model mice proved that oxidative stress played a crucial role in lipid dysregulation and peroxidation at the molecular level. Lipid hydroperoxides, being involved in the vicious circle of the lipid abnormal metabolism and energy production disturbance, showed the potential to be the possible diagnostic markers and the evaluation indexes for the effects of the antioxidants. As crucial metabolism organs, both liver and kidney expressed oxidative stress-involved lipidome dysregulation and, at the same time, suggested organ-specific characteristics. Our data provided a better understanding of oxidative stress in NASH development from the view of lipid metabolism, providing direct evidence for the oxidative damage-caused lipid hydroperoxidation in NASH.

## Data Availability

Data is contained within the article or supplementary material.

## Supplementary Materials

The following are available online at https://www.mdpi.com/article/10.3390/antiox10101602/s1.

## Abbreviations

AUC	Area under curve
CKD	Chronic kidney disease
CL	Cardiolipin
FFA	Free fatty acid
IS	Internal standard
LPC	Lysophosphatidylcholine
LPE	Lysophosphatidylethanolamine
LPI	Lysophosphatidylinositol
NAFLD	Nonalcoholic fatty liver disease
NASH	Nonalcoholic steatohepatitis
OPLS-DA	Orthogonal partial least squares discriminant analysis
PC	Phosphatidylcholine
PCA	Principal component analysis
PCOOH	Phosphatidylcholine hydroperoxide
PE	Phosphatidylethanolamine
PI	Phosphatidylinositol
PlsCho	Plasmalogen choline
PlsEtn	Plasmalogen ethanolamine
PUFA	Polyunsaturated fatty acid
ROS	Reactive oxygen species
TG	Triacylglycerol
TGOOH	Triacylglycerol hydroperoxide
