# Supplementary material for: Oxidative Stress Linked Organ Lipid Hydroperoxidation and Dysregulation in Mouse Model of Nonalcoholic Steatohepatitis: Revealed by Lipidomic Profiling of Liver and Kidney
Source: Antioxidants (Basel). 2021 Oct 12;10(10):1602. doi: 10.3390/antiox10101602 (PMC8533338; doi:10.3390/antiox10101602)
Supplement: Supplementary file 1 [file antioxidants-10-01602-s001.zip › antioxidants-1381076-supple material 1 .pdf]

Supplementary Material 1

(The detailed information of animal feeding and modeling)

Four-week-old male mice (C57BL/6J) were purchased from Charles River Japan (Yokohama, Japan) and fed a normal chow (Oriental Yeast Co., Ltd., Tokyo, Japan) during a 7-day acclimatization period ad libitum. After acclimatization, these mice were randomly divided into the normal group (n = 8) and the NASH model group (n = 8).

The normal group were fed on normal chow for 24 weeks with intravenous injections of PBS (0.2 mL per mouse, once every 3 days, 8 times) from 21 weeks. While the NASH group were fed on a high-fat diet (HFD-60, Oriental Yeast Co., Ltd.) with intravenous injections of oxidized low- density lipoproteins (0.2 mL per mouse, once every 3 days, 8 times) from 21 weeks. During the experiment, the mice were fed ad libitum.

At the end of the experiment, the mice were anaesthetized using diethyl ether and sacrificed according to the protocol of institutional Animal Care and Use Committee after fasting for 12 hours. According to our previous study, this mice-based model shows similar histopathological and metabolic features to human NASH.
