# Supplementary material for: Oxidative Stress Linked Organ Lipid Hydroperoxidation and Dysregulation in Mouse Model of Nonalcoholic Steatohepatitis: Revealed by Lipidomic Profiling of Liver and Kidney
Source: Antioxidants (Basel). 2021 Oct 12;10(10):1602. doi: 10.3390/antiox10101602 (PMC8533338; doi:10.3390/antiox10101602)
Supplement: Supplementary file 1 [file antioxidants-10-01602-s001.zip › antioxidants-1381076-supple material 2.pdf]

Supplementary Material 2

Table S1. Identification of the intact lipids detected in liver and kidney samples

Lipid species	RT (min)	Ion	Calc. (*m/z*)	Test (*m/z*)	Δppm	MS/MS signals	Molecule species	Exitance
TG								
42:0	13.31	$[M+NH_4]^+$	740.6763	740.6769	0.81			L, K
44:0	13.72	$[M+NH_4]^+$	768.7076	768.7086	1.30			L, K
46:0	14.13	$[M+NH_4]^+$	796.7389	796.7393	0.50			L, K
46:1	13.79	$[M+NH_4]^+$	794.7232	794.7239	0.88	549,523,521	14:0/16:1/16:0	L, K
46:2	13.55	$[M+NH_4]^+$	792.7076	792.7085	1.14	549,521,519	14:1/16:1/16:0	L, K
46:3	13.16	$[M+NH_4]^+$	790.6919	790.6921	0.25	547,519	14:1/16:1/16:1	L, K
48:0	14.47	$[M+NH_4]^+$	824.7702	824.7705	0.36			L, K
48:1	14.17	$[M+NH_4]^+$	822.7545	822.7551	0.73	549,551<br>577,523,549	16:0/16:1/16:0<br>14:0/18:1/16:0	L, K
48:2	13.88	$[M+NH_4]^+$	820.7389	820.7396	0.85	547,549<br>575,521,549	16:0/16:1/16:1<br>14:0/18:1/16:1	L, K
48:3	13.59	$[M+NH_4]^+$	818.7232	818.7228	-0.49	547<br>573,521,547	16:1/16:1/16:1<br>14:0/18:2/16:1	L, K
48:4	13.27	$[M+NH_4]^+$	816.7076	816.7062	-1.71	545,547	16:1/16:2/16:1<br>14:1/16:1/18:2	L, K
48:5	13.20	$[M+NH_4]^+$	814.6919	814.6890	-3.56	543,547	16:1/16:3/16:1	L, K
50:0	14.85	$[M+NH_4]^+$	852.8015	852.8026	1.29			L, K
50:1	14.49	$[M+NH_4]^+$	850.7858	850.7853	-0.59	577,551,577	16:0/18:1/16:0	L, K
50:2	14.26	$[M+NH_4]^+$	848.7702	848.7703	0.12	575,549,577	16:0/18:1/16:1	L, K
50:3	13.97	$[M+NH_4]^+$	846.7545	846.7548	0.35	575,547	16:1/18:1/16:1	L, K
50:4	13.66	$[M+NH_4]^+$	844.7389	844.7385	-0.47	573,547	16:1/18:2/16:1	L, K
50:5	13.55	$[M+NH_4]^+$	842.7232	842.7220	-1.42	573,571,545	16:2/18:2/16:1	L, K
50:6	13.28	$[M+NH_4]^+$	840.7076	840.7056	-2.38	573,569,543	16:3/18:2/16:1	L, K
52:0	15.28	$[M+NH_4]^+$	880.8328	880.8329	0.11			L, K
52:1	14.92	$[M+NH_4]^+$	878.8171	878.8173	0.23	605,579,577	16:0/18:1/18:0	L, K
52:2	14.58	$[M+NH_4]^+$	876.8015	876.8007	-0.91	603,577	16:0/18:1/18:1	L, K
52:3	14.33	$[M+NH_4]^+$	874.7858	874.7855	-0.34	603,575<br>601,577,575	16:1/18:1/18:1<br>16:0/18:2/18:1	L, K
52:4	13.99	$[M+NH_4]^+$	872.7702	872.7691	-1.26	601,575,573	16:1/18:2/18:1	L, K
52:5	13.80	$[M+NH_4]^+$	870.7545	870.7536	-1.03	599,573	16:1/18:2/18:2	L, K
52:6	13.73	$[M+NH_4]^+$	868.7389	868.7390	0.12	597,573,571	16:1/18:3/18:2	L, K
52:7	13.31	$[M+NH_4]^+$	866.7232	866.7232	0.00	595,571	16:1/18:3/18:3<br>16:2/18:3/18:2	L, K
52:8	13.22	$[M+NH_4]^+$	864.7076	864.7072	-0.46	595,569	16:2/18:3/18:3	L, K
54:0	15.73	$[M+NH_4]^+$	908.8641	908.8640	-0.11			L, K
54:1	15.34	$[M+NH_4]^+$	906.8484	906.8483	-0.11	607,605<br>633,607,577	18:0/18:1/18:0<br>16:0/18:1/20:0	L, K
54:2	14.99	$[M+NH_4]^+$	904.8328	904.8323	-0.55	605,603<br>631,605,577	18:0/18:1/18:1<br>16:0/18:1/20:1	L, K
54:3	14.70	$[M+NH_4]^+$	902.8171	902.8160	-1.22	603<br>629,603,577	18:1/18:1/18:1<br>16:0/18:1/20:2	L, K
54:4	14.40	$[M+NH_4]^+$	900.8015	900.8001	-1.55	603,601<br>627,601,577	18:1/18:2/18:1<br>16:0/18:1/20:3	L, K
54:5	14.17	$[M+NH_4]^+$	898.7858	898.7820	-4.23	599,601<br>625,601,575	18:1/18:2/18:2<br>16:0/18:2/20:3	L, K
54:6	13.91	$[M+NH_4]^+$	896.7702	896.7692	-1.12	623,599,575	16:0/18:2/20:4	L, K
54:7	13.82	$[M+NH_4]^+$	894.7545	894.7543	-0.22	621,599,573	16:0/18:3/20:4	L, K

54:8	13.57	$[M+NH_4]^+$	892.7389	892.7390	0.11	621,597,571<br>597,595	16:1/18:3/20:4<br>18:2/18:3/18:3	L, K
54:9	13.29	$[M+NH_4]^+$	890.7232	890.7228	-0.45	621,595,569<br>619,595,571	16:2/18:3/20:4<br>16:1/18:3/20:5	L, K
54:10	12.98	$[M+NH_4]^+$	888.7076	888.7070	-0.68			L, K
56:4	14.78	$[M+NH_4]^+$	928.8328	928.8307	-2.26	629,627,605<br>655,629,577	18:0/20:3/18:1<br>16:0/18:1/22:3	L, K
56:5	14.45	$[M+NH_4]^+$	926.8171	926.8146	-2.70			L, K
56:6	14.47	$[M+NH_4]^+$	924.8015	924.7999	-1.73			L, K
56:7	14.18	$[M+NH_4]^+$	922.7858	922.7849	-0.98	649,623,577	16:0/18:1/22:6	L, K
56:8	13.91	$[M+NH_4]^+$	920.7702	920.7694	-0.87	647,623,575	16:0/18:2/22:6	L, K
56:9	13.63	$[M+NH_4]^+$	918.7545	918.7540	-0.54	647,621,573	16:1/18:2/22:6	L, K
56:10	13.33	$[M+NH_4]^+$	916.7389	916.7380	-0.98	647,619,571<br>645,621,571	16:2/18:2/22:6<br>16:1/18:3/22:6	L, K
56:11	13.22	$[M+NH_4]^+$	914.7232	914.7225	-0.77			L, K
58:6	14.81	$[M+NH_4]^+$	952.8328	952.8316	-1.26			L, K
58:7	14.45	$[M+NH_4]^+$	950.8171	950.8157	-1.47			L, K
58:8	14.26	$[M+NH_4]^+$	948.8015	948.8002	-1.37			L, K
58:9	13.97	$[M+NH_4]^+$	946.7858	946.7850	-0.84			L, K
58:10	13.70	$[M+NH_4]^+$	944.7702	944.7693	-0.95	647,599	18:2/18:2/22:6	L, K
58:11	13.61	$[M+NH_4]^+$	942.7545	942.7538	-0.74	647,645,597	18:2/18:3/22:6	L, K
58:12	13.29	$[M+NH_4]^+$	940.7389	940.7386	-0.32			L, K
58:13	13.12	$[M+NH_4]^+$	938.7232	938.7223	-0.96			L, K
60:10	14.13	$[M+NH_4]^+$	972.8015	972.8006	-0.93			L, K
60:11	13.93	$[M+NH_4]^+$	970.7858	970.7849	-0.93			L, K
60:12	13.77	$[M+NH_4]^+$	968.7702	968.7692	-1.03			L, K
60:13	13.49	$[M+NH_4]^+$	966.7545	966.7543	-0.21			L, K
60:14	13.20	$[M+NH_4]^+$	964.7389	964.7384	-0.52			L, K
60:15	13.00	$[M+NH_4]^+$	962.7232	962.7233	0.10			L, K
62:12	14.00	$[M+NH_4]^+$	996.8015	996.8006	-0.90			L, K
64.17	13.24	$[M+NH_4]^+$	1014.7545	1014.7538	-0.69			L, K
66:18	13.40	$[M+NH_4]^+$	1040.7702	1040.7704	0.19			L, K
FFA								
14:0	7.75	$[M-H]^-$	227.2017	227.2019	0.88	NA	NA	L, K
14:1	6.03	$[M-H]^-$	225.1860	225.1863	1.33	NA	NA	L, K
16:0	10.04	$[M-H]^-$	255.2330	255.2330	0.00	NA	NA	L, K
16:1	8.51	$[M-H]^-$	253.2173	253.2175	0.79	NA	NA	L, K
16:2	6.95	$[M-H]^-$	251.2017	251.2020	1.19	NA	NA	L, K
16:3	5.81	$[M-H]^-$	249.1860	249.1864	1.61	NA	NA	L, K
18:0	11.82	$[M-H]^-$	283.2643	283.2644	0.35	NA	NA	L, K
18:1	10.62	$[M-H]^-$	281.2486	281.2489	1.07	NA	NA	L, K
18:2	9.42	$[M-H]^-$	279.2330	279.2332	0.72	NA	NA	L, K
18:3	8.17	$[M-H]^-$	277.2173	277.2178	1.80	NA	NA	L, K
20:0	13.32	$[M-H]^-$	311.2956	311.2959	0.96	NA	NA	L, K
20:1	12.04	$[M-H]^-$	309.2799	309.2801	0.65	NA	NA	L, K
20:2	10.97	$[M-H]^-$	307.2643	307.2646	0.98	NA	NA	L, K
20:3	9.98	$[M-H]^-$	305.2486	305.2488	0.66	NA	NA	L, K

20:4	9.37	[M-H]-	303.2330	303.2334	1.32	NA	NA	L, K
20:5	8.20	[M-H]-	301.2173	301.2175	0.66	NA	NA	L, K
22:6	9.10	[M-H]-	327.2330	327.2331	0.31	NA	NA	L, K
PC								
28:0	7.97	[M+H]+	678.5068	678.5073	0.74			L, K
30:0	8.51	[M+H]+	706.5381	706.5375	-0.85			L, K
30:1	8.06	[M+H]+	704.5225	704.5215	-1.42			L, K
32:0	9.18	[M+H]+	734.5694	734.5696	0.27	478	16:0/16:0	L, K
32:1	8.60	[M+H]+	732.5538	732.5538	0.00			L, K
32:2	8.14	[M+H]+	730.5381	730.5378	-0.41			L, K
32:3	7.58	[M+H]+	728.5225	728.5223	-0.27			L, K
34:0	10.52	[M+H]+	762.6007	762.6010	0.39			L, K
34:1	9.41	[M+H]+	760.5851	760.5850	-0.13	478,504	16:0/18:1	L, K
34:2	8.88	[M+H]+	758.5694	758.5685	-1.19	504,494	16:1/18:1	L, K
34:2	9.10	[M+H]+	758.5694	758.5693	-0.13	420,502,496,478	16:0/18:2	L
34:3	8.08	[M+H]+	756.5538	756.5536	-0.26	520,502,494,476	16:0/18:3	L, K
34:3	8.37	[M+H]+	756.5538	756.5554	2.11	518,500,496,478	16:1/18:2	L, K
34:4	8.06	[M+H]+	754.5381	754.5381	0.00			L, K
34:5	7.75	[M+H]+	752.5225	752.5220	-0.66			L, K
36:0	11.72	[M+H]+	790.6320	790.6351	3.92			K
36:1	10.44	[M+H]+	788.6164	788.6165	0.13	506,504	18:0/18:1	L, K
36:2	9.86	[M+H]+	786.6007	786.6006	-0.13	506,502	18:0/18:2	L, K
36:3	9.02	[M+H]+	784.5851	784.5854	0.38	504,502	18:1/18:2	L, K
36:4	8.37	[M+H]+	782.5694	782.5677	-2.17	520,502	18:2/18:2	L, K
36:4	8.57	[M+H]+	782.5694	782.5684	-1.28	546,528,494,476	16:1/20:3	L, K
36:4	9.06	[M+H]+	782.5694	782.5691	-0.38	544,526,496,478	16:0/20:4	L
36:5	8.08	[M+H]+	780.5538	780.5533	-0.64	520,502,518,500	18:2/18:3	L, K
36:5	8.25	[M+H]+	780.5538	780.5533	-0.64	542,524,496,478	16:0/20:5	L, K
36:6	7.97	[M+H]+	778.5381	778.5380	-0.13	568,550,468,450	14:0/22:6	L, K
38:4	9.83	[M+H]+	810.6007	810.5999	-0.99	544,526,524,506	18:0/20:4	L, K
38:5	8.98	[M+H]+	808.5851	808.5832	-2.35	544,526,522,504	18:1/20:4	L, K
38:6	8.49	[M+H]+	806.5694	806.5688	-0.74	568,550,496,478	16:0/22:6	L, K
38:6	8.60	[M+H]+	806.5694	806.5682	-1.49			L, K
38:6	9.07	[M+H]+	806.5694	806.5682	-1.49			L, K
38:7	8.10	[M+H]+	804.5538	804.5533	-0.62	568,550,494,476	16:1/22:6	L, K
38:8	7.90	[M+H]+	802.5381	802.5380	-0.12	568,550,492,474	16:2/22:6	L, K
40:6	9.56	[M+H]+	834.6007	834.5992	-1.80	568,550,524,506	18:0/22:6	L, K
40:7	8.80	[M+H]+	832.5851	832.5850	-0.12	522,504,550,568	18:1/22:6	L, K
40:7	9.03	[M+H]+	832.5851	832.5828	-2.76	520,502,552,570	18:2/22:5	L
40:8	8.28	[M+H]+	830.5694	830.5684	-1.20			L, K
40:9	7.99	[M+H]+	828.5538	828.5529	-1.09			L, K
40:10	7.80	[M+H]+	826.5381	826.5374	-0.85			L, K
42:10	8.17	[M+H]+	854.5694	854.5678	-1.87	568,550,544,526	20:4/22:6	L, K
42:11	7.85	[M+H]+	852.5538	852.5532	-0.70			L, K

44:12	8.10	$[M+H]^+$	878.5694	878.5684	-1.14			L, K
PlsCho								
34:0	17.60	$[M+CH_3COO]^-$	804.6124	804.6125	0.12	730,464,283	p16:0/18:0	L, K
34:1	17.07	$[M+CH_3COO]^-$	802.5967	802.5984	2.12	728,464,281	p16:0/18:1	K
34:3	16.40	$[M+CH_3COO]^-$	798.5654	798.5659	0.63			K
34:4	14.95	$[M+CH_3COO]^-$	796.5498	796.5487	-1.38			K
34:5	14.59	$[M+CH_3COO]^-$	794.5341	794.5330	-1.38			L, K
36:3	17.11	$[M+CH_3COO]^-$	826.5967	826.5961	-0.73			L, K
36:4	17.03	$[M+CH_3COO]^-$	824.5811	824.5811	0.00	750,464,303	p16:0/20:4	K
36:5	16.26	$[M+CH_3COO]^-$	822.5654	822.5668	1.70	748,464,301	p16:0/20:5	K
36:5	16.70	$[M+CH_3COO]^-$	822.5654	822.5668	1.70	748,462,303	p16:1/20:4	K
38:4	17.50	$[M+CH_3COO]^-$	852.6124	852.6137	1.52	778,492,303	p18:0/20:4	L, K
38:5	17.03	$[M+CH_3COO]^-$	850.5967	850.5969	0.24	776,490,303	p18:1/20:4	K
38:6	16.42	$[M+CH_3COO]^-$	848.5811	848.5807	-0.47	774,464,327	p16:0/22:6	K
38:7	16.19	$[M+CH_3COO]^-$	846.5654	846.5661	0.83			K
40:6	17.25	$[M+CH_3COO]^-$	876.6124	876.6133	1.03	802,492,327	p18:0/22:6	K
40:7	17.10	$[M+CH_3COO]^-$	874.5967	874.5972	0.57			K
LPC								
14:0	2.97	$[M+H]^+$	468.3085	468.3083	-0.43	NA	NA	L, K
16:0	3.39	$[M+H]^+$	496.3398	496.3395	-0.60	NA	NA	L, K
16:1	2.88	$[M+H]^+$	494.3241	494.3243	0.40	NA	NA	L, K
18:0	4.47	$[M+H]^+$	524.3711	524.3705	-1.14	NA	NA	L, K
18:1	3.40	$[M+H]^+$	522.3554	522.3549	-0.96	NA	NA	L, K
18:2	2.95	$[M+H]^+$	520.3398	520.3398	0.00	NA	NA	L, K
18:3	2.78	$[M+H]^+$	518.3241	518.3242	0.19	NA	NA	L, K
20:4	2.89	$[M+H]^+$	544.3398	544.3396	-0.37	NA	NA	L, K
20:5	2.69	$[M+H]^+$	542.3241	542.3239	-0.37	NA	NA	L, K
22:6	2.87	$[M+H]^+$	568.3398	568.3393	-0.88	NA	NA	L, K
PE								
32:1	16.59	$[M-H]^-$	688.4923	688.4938	2.18			L, K
32:2	15.95	$[M-H]^-$	686.4766	686.4793	3.93			L, K
34:0	17.85	$[M-H]^-$	718.5392	718.5400	1.11			L, K
34:1	17.28	$[M-H]^-$	716.5236	716.5258	3.07	452,434,281	16:0/18:1	L, K
34:2	16.79	$[M-H]^-$	714.5079	714.5089	1.40	452,279,255	16:0/18:2	L, K
34:3	16.22	$[M-H]^-$	712.4923	712.4933	1.40			L, K
34:4	15.86	$[M-H]^-$	710.4766	710.4775	1.27			L, K
36:1	17.99	$[M-H]^-$	744.5549	744.5577	3.76	462,480,281	18:0/18:1	L, K
36:2	17.51	$[M-H]^-$	742.5392	742.5427	4.71			L, K
36:3	16.21	$[M-H]^-$	740.5236	740.5270	4.59	478,279,281	18:1/18:2	L, K
36:4	16.53	$[M-H]^-$	738.5079	738.5115	4.87	279,476	18:2/18:2	L, K
36:4	16.96	$[M-H]^-$	738.5079	738.5107	3.79	255,303,452	16:0/20:4	L, K
36:5	16.25	$[M-H]^-$	736.4923	736.4954	4.21	450,303,253	16:1/20:4	L, K

36:5	16.44	$[M-H]^-$	736.4923	736.4954	4.21	452,301,255	16:0/20:5	L, K
36:6	15.97	$[M-H]^-$	734.4766	734.4799	4.49	424,406,327	14:0/22:6	L, K
38:4	17.67	$[M-H]^-$	766.5392	766.5423	4.04	480,303,283	18:0/20:4	L, K
38:5	16.70	$[M-H]^-$	764.5236	764.5270	4.45	478,303,281	18:1/20:4	L, K
38:5	16.90	$[M-H]^-$	764.5236	764.5270	4.45	480,462,301	18:0/20:5	L, K
38:6	16.79	$[M-H]^-$	762.5079	762.5105	3.41	478,301,281	18:1/20:5	L, K
38:7	16.10	$[M-H]^-$	760.4923	760.4960	4.87	524,450,327,253	16:1/22:6	L, K
38:7	16.44	$[M-H]^-$	760.4923	760.4957	4.47	522,452,325,255	16:0/22:7	L, K
38:8	15.91	$[M-H]^-$	758.4766	758.4794	3.69			L, K
40:6	17.58	$[M-H]^-$	790.5392	790.5428	4.55	480,327,283	18:0/22:6	L, K
40:8	16.31	$[M-H]^-$	786.5079	786.5112	4.20	476,327,279	18:2/22:6	L, K
40:8	16.66	$[M-H]^-$	786.5079	786.5114	4.45	482,303	20:4/20:4	L, K
40:9	16.03	$[M-H]^-$	784.4923	784.4948	3.19	474,456,327	18:3/22:6	L, K
40:10	15.54	$[M-H]^-$	782.4766	782.4730	-4.60	498,480,301	20:5/20:5	L, K
42:10	16.34	$[M-H]^-$	810.5079	810.5093	1.73	524,506,303	20:4/22:6	L, K
42:11	15.92	$[M-H]^-$	808.4923	808.4919	-0.49	283,437	20:5/22:6	L, K
44:12	16.31	$[M-H]^-$	834.5079	834.5076	-0.36	327,463	22:6/22:6	L, K
PlsEtn								
32:1	16.94	$[M-H]^-$	672.4974	672.4982	1.19	436,418,253	p16:0/16:1	
34:0	18.15	$[M-H]^-$	702.5443	702.5444	0.14			L, K
34:1	17.63	$[M-H]^-$	700.5287	700.5284	-0.43	436,418,281	p16:0/18:1	L, K
34:2	17.17	$[M-H]^-$	698.5130	698.5134	0.57	436,418,279	p16:0/18:2	L, K
34:3	16.60	$[M-H]^-$	696.4974	696.4986	1.72			K
34:4	16.42	$[M-H]^-$	694.4817	694.4819	0.29	408,390,303	p14:0/20:4	K
36:0	18.80	$[M-H]^-$	730.5756	730.5756	0.00			K
36:1	18.28	$[M-H]^-$	728.5600	728.5598	-0.27	446,464,281	p18:0/18:1	L, K
36:2	17.86	$[M-H]^-$	726.5443	726.5442	-0.14	462,444,281	p18:1/18:1	L, K
36:3	17.32	$[M-H]^-$	724.5287	724.5290	0.41			L, K
36:4	17.19	$[M-H]^-$	722.5130	722.5135	0.69	436,418,303	p16:0/20:4	L, K
36:5	16.78	$[M-H]^-$	720.4974	720.4977	0.42	436,418,303	p16:0/20:5	L, K
36:6	16.33	$[M-H]^-$	718.4817	718.4828	1.53	408,390,327	p14:0/22:6	L, K
38:4	17.89	$[M-H]^-$	750.5443	750.5435	-1.07	464,446,303	p18:0/20:4	L, K
38:5	17.33	$[M-H]^-$	748.5287	748.5278	-1.20	462,444,303	p18:1/20:4	L, K
38:5	17.59	$[M-H]^-$	748.5287			436,418,329	p16:0/22:5	L, K
38:6	17.17	$[M-H]^-$	746.5130	746.5129	-0.13	436,418,327	p16:0/22:6	L, K
38:7	16.60	$[M-H]^-$	744.4974	744.4989	2.01	434,416,327	p16:1/22:6	L, K
40:6	17.83	$[M-H]^-$	774.5443	774.5457	1.81	464,446,327	p18:0/22:6	L, K
40:7	17.27	$[M-H]^-$	772.5287	772.5292	0.65	462,444,327	p18:1/22:6	L, K
40:8	16.82	$[M-H]^-$	770.5130	770.5132	0.26	460,442,327	p18:2/22:6	L, K
40:9	16.48	$[M-H]^-$	768.4974	768.4980	0.78			K
40:10	15.91	$[M-H]^-$	766.4817	766.4837	2.61			L, K
42:10	16.62	$[M-H]^-$	794.5130	794.5129	-0.13			K
LPE								

16:0	9.84	[M-H]-	452.2783	452.2799	3.54	NA	NA	L, K
16:1	8.38	[M-H]-	450.2626	450.2646	4.44	NA	NA	L, K
18:0	11.12	[M-H]-	480.3096	480.3098	0.42	NA	NA	L, K
18:1	10.34	[M-H]-	478.2939	478.2952	2.72	NA	NA	L, K
1-18:2	8.82	[M-H]-	476.2783	476.2786	0.63	NA	NA	L, K
2-18:2	9.24	[M-H]-	476.2783	476.2787	0.84	NA	NA	L, K
20:4	8.88	[M-H]-	500.2783	500.2798	3.00	NA	NA	L, K
20:5	7.76	[M-H]-	498.2626	498.2642	3.21	NA	NA	L, K
22:6	8.95	[M-H]-	524.2783	524.2799	3.05	NA	NA	L, K
PI								
30:0	13.53	[M-H]-	781.4873	781.4869	-0.51			L, K
32:0	14.24	[M-H]-	809.5186	809.5174	-1.48			L, K
32:1	13.56	[M-H]-	807.5029	807.5031	0.25			L, K
32:2	13.58	[M-H]-	805.4873	805.4887	1.74			L, K
34:0	14.67	[M-H]-	837.5499	837.5496	-0.36			L, K
34:1	14.16	[M-H]-	835.5342	835.5350	0.96	255,281,391,417,553,579	16:0/18:1	L, K
34:1	14.46	[M-H]-	835.5342	835.5323	-2.27	253,283,389,419,551,581	16:1/18:0	L, K
34:2	13.76	[M-H]-	833.5186	833.5208	2.64	255,279,391,415,595	16:0/18:2	L, K
34:2	14.07	[M-H]-	833.5186	833.5197	1.32	253,281,389,417,569	16:1/18:1	L, K
34:3	13.31	[M-H]-	831.5029	831.5036	0.84			L, K
34:4	13.19	[M-H]-	829.4873	829.4878	0.60			L, K
36:0	16.34	[M-H]-	865.5812	865.5792	-2.31	283,419,599	18:0/18:0	L, K
36:1	14.95	[M-H]-	863.5655	863.5674	2.20			L, K
36:2	14.41	[M-H]-	861.5499	861.5511	1.39			L, K
36:3	14.04	[M-H]-	859.5342	859.5361	2.21			L, K
36:4	13.83	[M-H]-	857.5186	857.5197	1.28	255,303,391,553,571	16:0/20:4	L, K
36:5	13.52	[M-H]-	855.5029	855.5046	1.99	255,301,391,437,571	16:0/20:5	L, K
36:6	13.09	[M-H]-	853.4873	853.4894	2.46	253,301,389,437,569	16:1/20:5	L, K
38:4	14.46	[M-H]-	885.5499	885.5496	-0.34	283,303,419,439,599	18:0/20:4	L, K
38:5	14.00	[M-H]-	883.5342	883.5344	0.23	281,303,417,439,597	18:1/20:4	L, K
38:5	14.13	[M-H]-	883.5342	883.5337	-0.57	255,329,391,465,571	16:0/22:5	L, K
38:6	13.69	[M-H]-	881.5186	881.5214	3.18	281,301,417,437,597	18:1/20:5	L, K
38:6	13.86	[M-H]-	881.5186	881.5209	2.61	255,283,391,463,571	16:0/22:6	L, K
38:7	13.50	[M-H]-	879.5029	879.5035	0.68			L, K
40:6	14.42	[M-H]-	909.5499	909.5492	-0.77	283,327,419,463,599	18:0/22:6	L, K
40:7	13.94	[M-H]-	907.5342	907.5338	-0.44	281,327,417,463,597	18:1/22:6	L, K
40:8	13.52	[M-H]-	905.5186	905.5189	0.33			L, K
42:10	13.50	[M-H]-	929.5186	929.5169	-1.83			K
LPI								
16:1	6.07	[M-H]-	569.2732	569.2721	-1.93	NA	NA	L, K
18:0	8.90	[M-H]-	599.3202	599.3209	1.17	NA	NA	L, K
18:1	7.62	[M-H]-	597.3045	597.3054	1.51	NA	NA	L, K
18:2	5.95	[M-H]-	595.2889	595.2891	0.34	NA	NA	L, K

20:4	5.97	$[M-H]^-$	619.2889	619.2896	1.13	NA	NA	L, K
22:6	5.97	$[M-H]^-$	643.2889	643.2897	1.24	NA	NA	K
CL								
68:2	18.24	$[M-H]^-$	1403.9962	1403.9947	-1.07	255,281	$(16:0)_2(18:1)_2$	L, K
68:3	18.03	$[M-H]^-$	1401.9806	1401.9807	0.07	253,255,281	$(16:0)_1(16:1)_1(18:1)_2$	L, K
68:4	17.85	$[M-H]^-$	1399.9649	1399.9629	-1.43			L
68:5	17.54	$[M-H]^-$	1397.9493	1397.9502	0.64			L, K
68:6	17.23	$[M-H]^-$	1395.9336	1395.9326	-0.72			L
70:4	18.10	$[M-H]^-$	1427.9962	1427.9940	-1.54	253,281	$(16:1)_1(18:1)_3$	L, K
70:5	17.84	$[M-H]^-$	1425.9806	1425.9834	1.96	253,279,281	$(16:1)_1(18:1)_2(18:2)_1$	L, K
70:6	17.72	$[M-H]^-$	1423.9649	1423.9628	-1.47			L, K
70:7	17.51	$[M-H]^-$	1421.9493	1421.9481	-0.84			L, K
70:8	17.44	$[M-H]^-$	1419.9336	1419.9315	-1.48			L
72:5	18.09	$[M-H]^-$	1454.0119	1454.0116	-0.21	279,281	$(18:1)_3(18:2)_1$	K
72:6	17.95	$[M-H]^-$	1451.9962	1451.9950	-0.83	279,281	$(18:1)_2(18:2)_2$	L, K
72:7	17.77	$[M-H]^-$	1449.9806	1449.9809	0.21	279,281	$(18:1)_1(18:2)_3$	L, K
72:8	17.58	$[M-H]^-$	1447.9649	1447.9671	1.52	279	$(18:2)_4$	L, K
72:9	17.11	$[M-H]^-$	1445.9493	1445.9473	-1.38			L, K
72:10	16.98	$[M-H]^-$	1443.9336	1443.9321	-1.04			L
74:7	17.97	$[M-H]^-$	1478.0119	1478.0122	0.20			K
74:8	17.87	$[M-H]^-$	1475.9962	1475.9965	0.20			L, K
74:9	17.78	$[M-H]^-$	1473.9806	1473.9818	0.81			L, K
74:10	17.69	$[M-H]^-$	1471.9649	1471.9636	-0.88			L, K
74:11	17.55	$[M-H]^-$	1469.9493	1469.9501	0.54			L
76:8	18.19	$[M-H]^-$	1504.0275	1504.0276	0.07			K
76:10	17.90	$[M-H]^-$	1499.9962	1499.9982	1.33			K
76:11	17.80	$[M-H]^-$	1497.9806	1497.9789	-1.13			L, K
76:12	17.55	$[M-H]^-$	1495.9649	1495.9635	-0.94			L, K

NA: not available; L, liver; and K, kidney.

Table S2. Identification of the hydroperoxides detected in liver and kidney samples

Lipid species	RT (min)	Ion	Calc. (*m/z*)	Test (*m/z*)	Δppm	Exitance
TGOOH						
52:2	12.63	$[M+NH_4]^+$	908.7913	908.7912	-0.11	L, K
52:3	12.05	$[M+NH_4]^+$	906.7756	906.7752	-0.44	L, K
52:4	11.51	$[M+NH_4]^+$	904.7600	904.7599	-0.11	L, K
54:2	13.02	$[M+NH_4]^+$	936.8226	936.8223	-0.32	L, K
54:3	12.68	$[M+NH_4]^+$	934.8069	934.8072	0.32	L, K
54:4	12.24	$[M+NH_4]^+$	932.7913	932.7906	-0.75	L, K
54:5	11.54	$[M+NH_4]^+$	930.7756	930.7761	0.54	L, K
58:10	10.78	$[M+NH_4]^+$	976.7600	976.7576	-2.46	K
58:11	10.33	$[M+NH_4]^+$	974.7443	974.7416	-2.77	K
PCOOH						
34:2	6.12	$[M+H]^+$	790.5593	790.5592	-0.13	L, K
34:3	5.61	$[M+H]^+$	788.5436	788.5439	0.38	L, K
36:4	5.98	$[M+H]^+$	814.5593	814.5602	1.10	L, K
36:5	5.44	$[M+H]^+$	812.5436	812.5436	0.00	L, K
36:6	3.83	$[M+H]^+$	810.5280	810.5281	0.12	L, K
38:5	6.13	$[M+H]^+$	840.5749	840.5745	-0.48	L, K
38:6	5.92	$[M+H]^+$	838.5593	838.5587	-0.72	L, K
38:7	5.08	$[M+H]^+$	836.5436	836.5432	-0.48	L, K
40:6	6.64	$[M+H]^+$	866.5906	866.5899	-0.81	L

L, liver; K, kidney.

A

Eigenvalues

Number	Eigenvalue	Percent	20 40 60 80	Cum Percent
1	116.2870	43.069		43.069
2	57.3502	21.241		64.310
3	26.1586	9.688		73.998
4	16.4391	6.089		80.087
5	10.4863	3.884		83.971
6	8.5253	3.158		87.128
7	5.3987	2.000		89.128
8	4.8063	1.780		90.908
9	3.6027	1.334		92.242
10	3.1131	1.153		93.395
11	2.7218	1.008		94.403
12	2.4411	0.904		95.307
13	1.8253	0.676		95.984
14	1.5469	0.573		96.556
15	1.1946	0.442		96.999
16	1.1082	0.410		97.409
17	0.9617	0.356		97.766
18	0.9194	0.341		98.106
19	0.7685	0.285		98.391
20	0.6479	0.240		98.631
21	0.5648	0.209		98.840
22	0.5375	0.199		99.039
23	0.4347	0.161		99.200
24	0.4143	0.153		99.353
25	0.3839	0.142		99.496
26	0.3632	0.135		99.630
27	0.2836	0.105		99.735
28	0.2283	0.085		99.820
29	0.1755	0.065		99.885
30	0.1653	0.061		99.946
31	0.1462	0.054		100.000

B

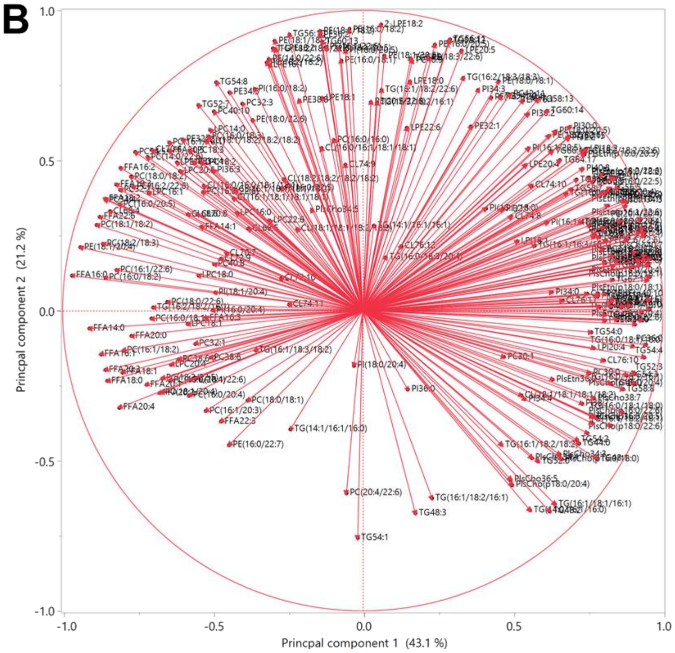

Figure S1. Detailed information of PCA. (**A**) Eigenvalues and (**B**) loading plot.

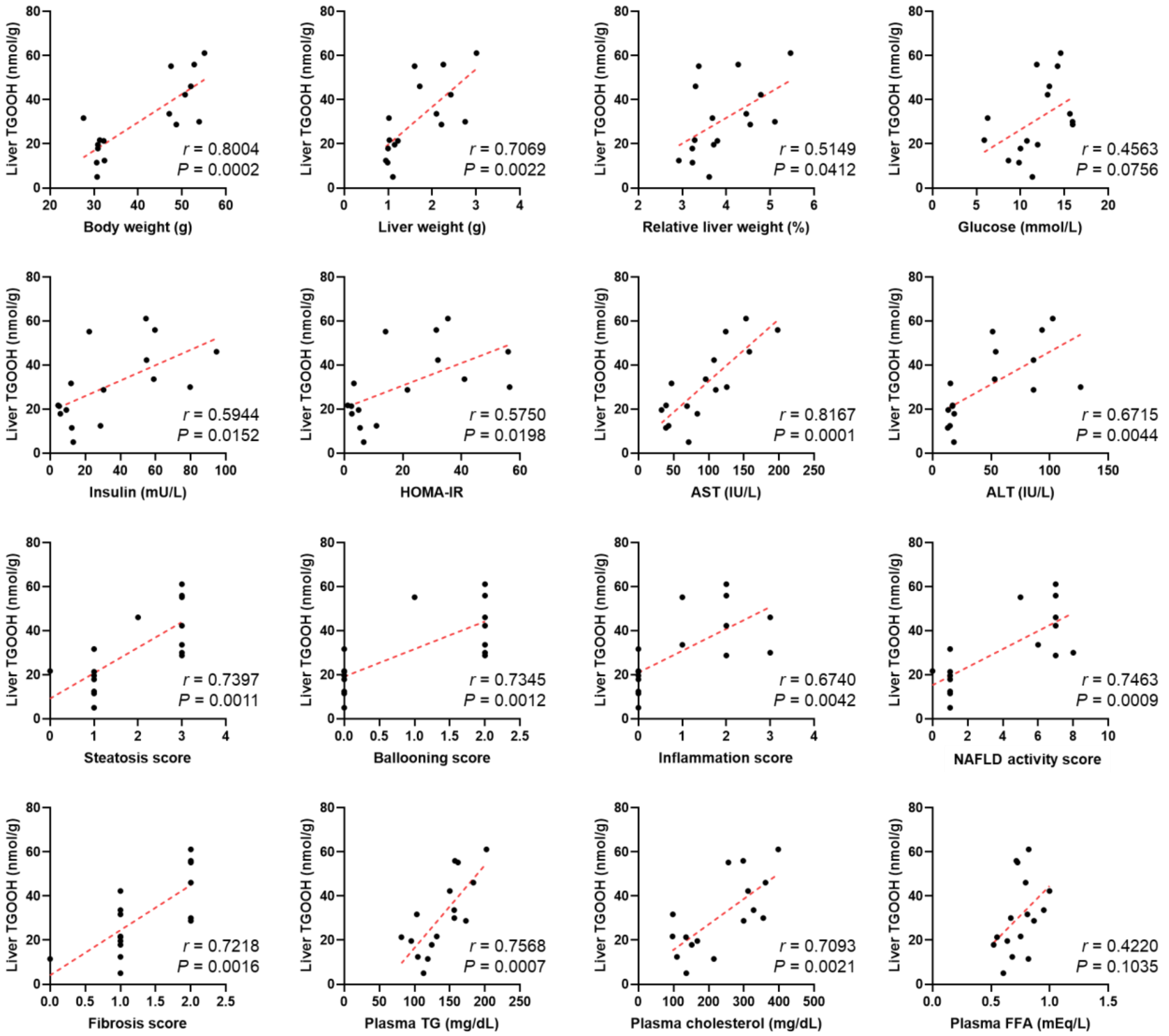

Figure S2. Correlation between total TGOOH content in liver and physiological indexes.

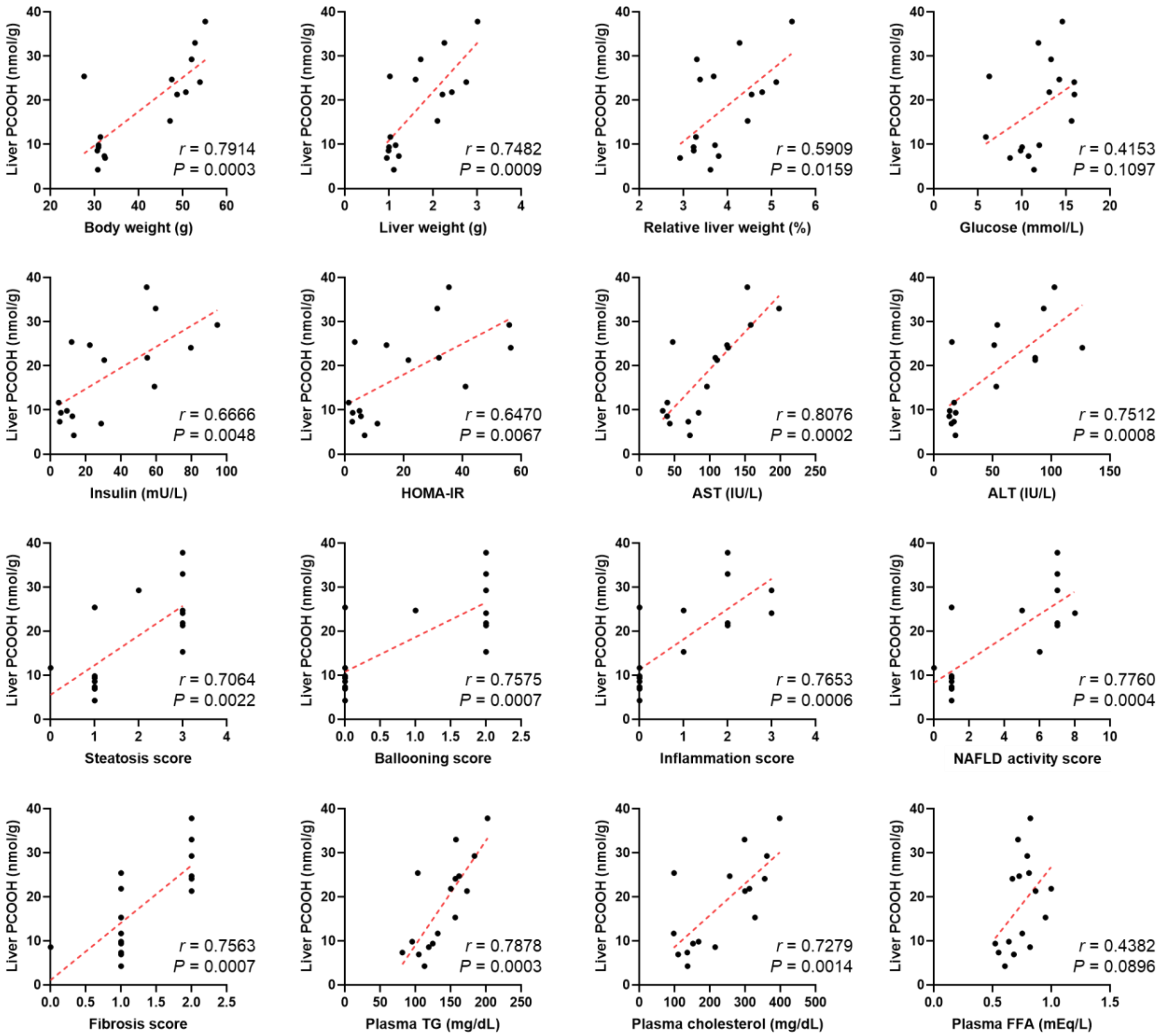

Figure S3. Correlation between total PCOOH content in liver and physiological indexes.

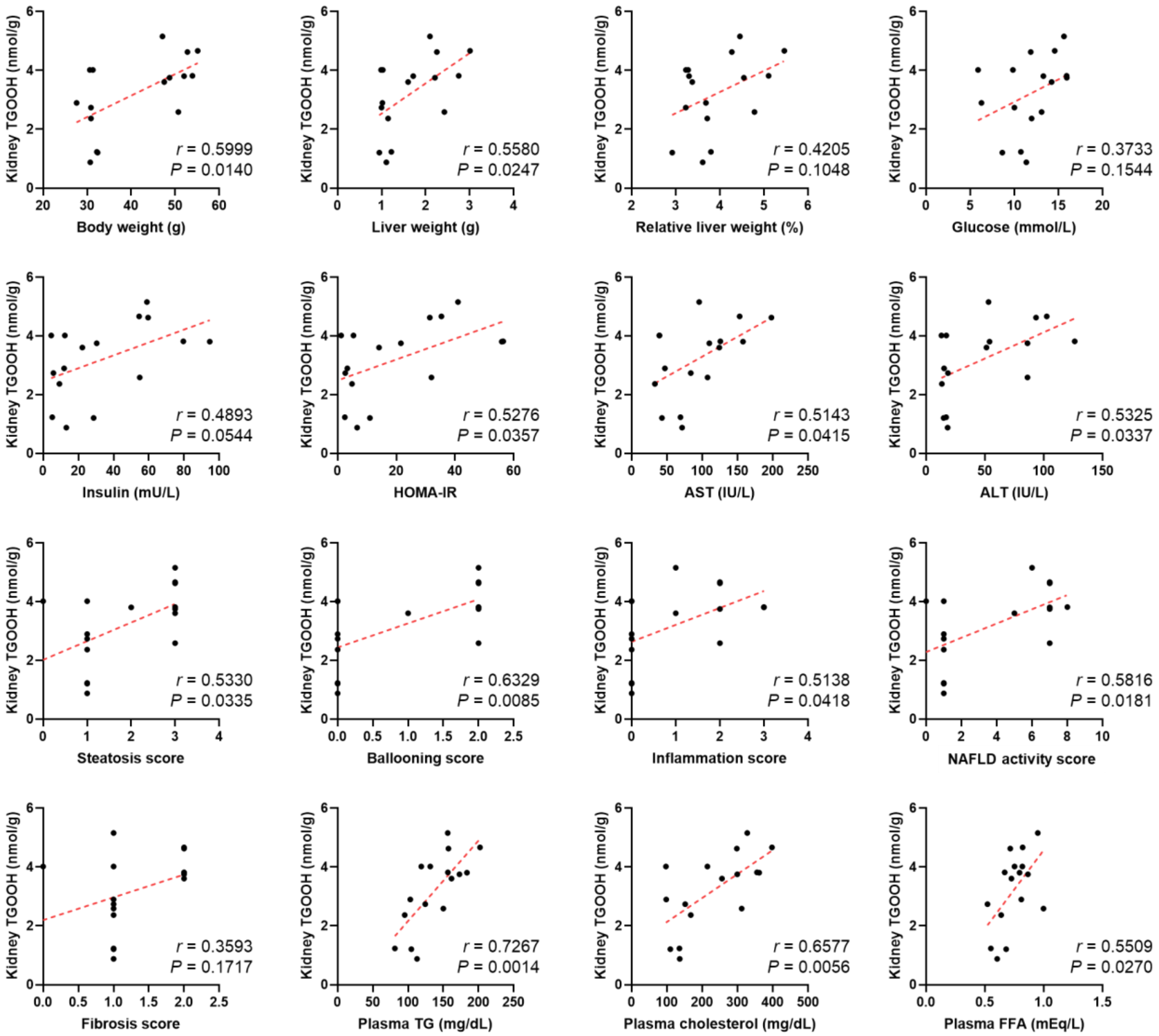

Figure S4. Correlation between total TGOOH content in kidney and physiological indexes.

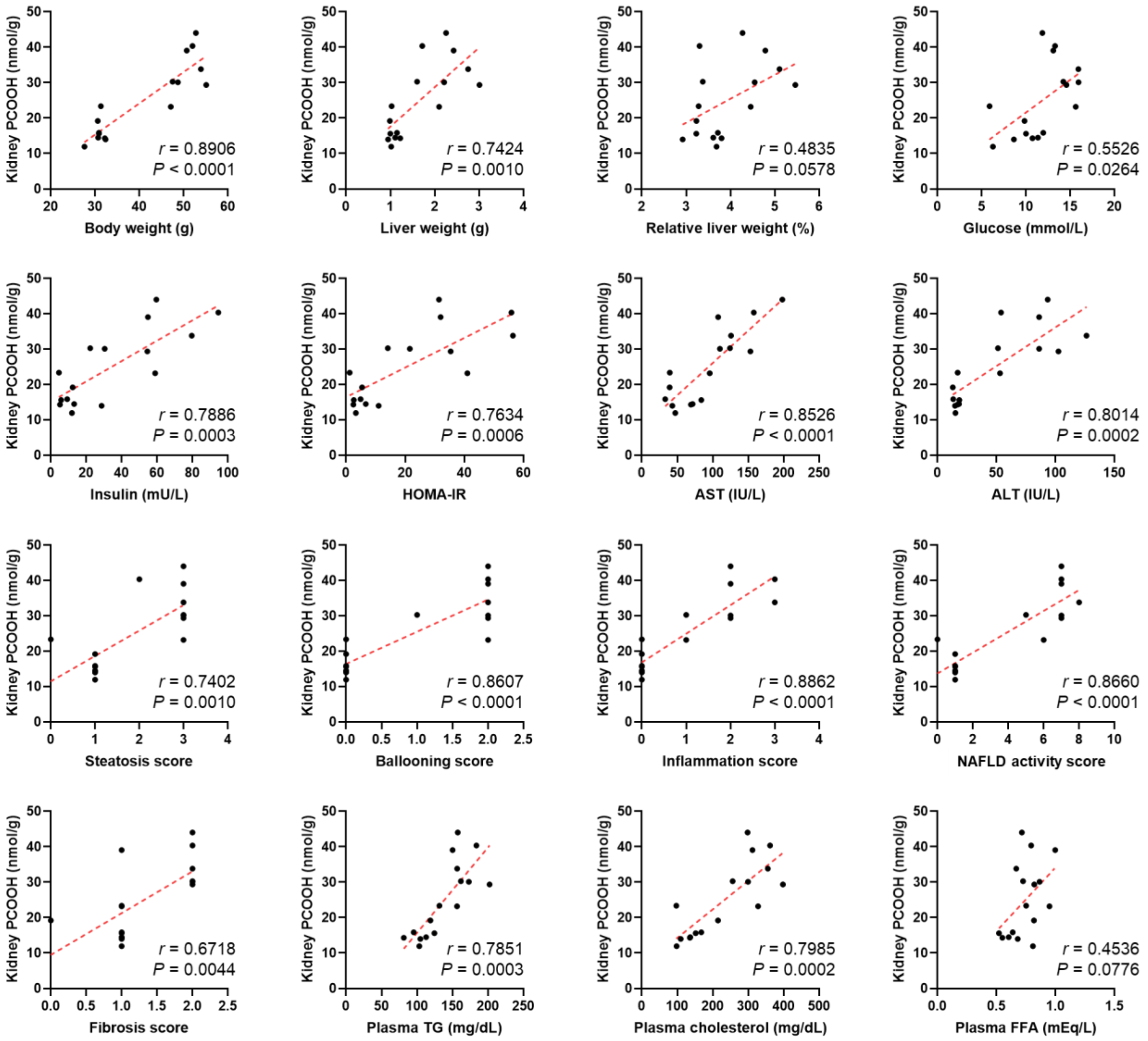

Figure S5. Correlation between total PCOOH content in kidney and physiological indexes.
